# Evaluation of the IncoStress device for urinary incontinence: a feasibility study and pilot randomised controlled trial

**DOI:** 10.1007/s00192-018-3749-5

**Published:** 2018-08-13

**Authors:** Hayser Medina Lucena, Kate Williams, Douglas G. Tincello, Allyson Lipp, Chris Shaw

**Affiliations:** 1 Department of Health Sciences, University of Leicester, Centre for Medicine, University Road, Leicester, Leicestershire LE1 7RH UK; 2 Faculty of Life Science and Education, University of South Wales, Pontypridd, Wales UK

**Keywords:** Acceptability, Effectiveness, Female, Pilot project, Treatment outcome, Vagina

## Abstract

**Introduction and hypothesis:**

The aim of this study was to assess the feasibility of recruitment to and outcomes from a pilot randomised study of the IncoStress device as an adjunct to conservative treatment for urinary incontinence.

**Methods:**

Women with urinary incontinence were randomised on a 2:1 basis to usual care (control) or usual care plus use of the IncoStress device (intervention). Process outcomes (retention and compliance) were recorded plus symptom outcomes (IQOL and ICIQ-FLUTS questionnaires). A sample of participants took part in an interview to understand the frequency of use of the device and satisfaction.

**Results:**

Eighty women (51 intervention, 29 control) were recruited. Follow-up responses were obtained from 34 intervention group (66.7%) and 17 (58.6%) control patients. Women used the device for a median 3 days a week (0–7), 18 out of 34 (53%) found it easy to use and 21 (61.8%) were satisfied with the device. Median IQOL score in the intervention group improved from a baseline of 42.4 (0–94) to 68.2 (5–98) at follow-up and in the control group from 45.5 (0–88) to 53.0 (0–94). Median ICIQ-FLUTS score in the intervention group improved from 14.5 (6–35) to 12.5 (4–26) and in the control group from 15.0 (5–35) to 14.0 (6–38).

**Conclusions:**

Recruitment and randomisation were feasible and robust. This study demonstrates that a large-scale RCT is feasible and the IncoStress has potential value.

## Introduction

Urinary incontinence (UI) in women is extremely common in middle and older age. Prevalence rates vary depending on sampling and definitions used; 49% of women over the age of 18 years have been reported to have incontinence [1]. Middle-aged and older women have a prevalence of between 25 and 40% [1].

In the UK the cost to the NHS for people with clinically significant urinary incontinence has been estimated at £536 million per annum and the patient-borne costs as £207 million per annum [2].

Treatments for UI include behavioural treatments (pelvic floor exercises, bladder training), which are resource intensive; pharmaceutical (anti-cholinergic medication for urge incontinence, duloxetine for stress incontinence); and also a range of surgical interventions [3]. Although there is also the opportunity for patients to use intraurethral or intravaginal mechanical devices, they are not consistently recommended by health care professionals. There is controversial evidence on the acceptability, compliance and satisfaction regarding the use of pessaries for urinary incontinence. Some studies reported no difference or greater satisfaction with behavioural therapy than with the use of pessaries [4, 5]. On the other hand recent studies have shown an increase in the acceptability and patients’ satisfaction [6–8] but it is to be noted that in some of these studies the pessaries were mainly fitted for pelvic organ prolapse with some participants having concomitant urinary symptoms.

The IncoStress is an intravaginal device designed to support the bladder neck and control mainly stress urinary incontinence. The manufacturer states that women with UUI and MUI may also benefit. Although there is no evidence, it has been marketed to help to strengthen pelvic floor muscles by stimulating contractions when holding the device in the vagina and also an added value of acting as a motivational aid. By giving women immediate improvements in symptoms it may help them to maintain motivation to persist with pelvic floor exercises. Motivation is key to compliance with behavioural treatments, and adherence to pelvic floor therapy is generally poor [9].

In general, there is minimal evidence on this type of device, so the role of this mechanical device in urinary incontinence needs further evaluation.

The purpose of this study was to examine the use of the IncoStress, assess recruitment and retention as well as acceptability of the device to patients, and use of potential outcome measures. Participants will be using the device in addition to their usual treatment (pelvic floor exercises) rather than as a replacement for usual care as pelvic floor exercises have proven efficacy.

## Methods and materials

This study was a mixed-methods feasibility study to inform the design of a potential larger randomised controlled trial. The study recruited women attending continence and physiotherapy services with different types of urinary incontinence. Ethical approval was obtained from the National Research Ethics Service in London-Stanmore (11/LO/0485).

Five centres from the UK were involved in the recruitment process. Women attending continence services were invited to participate and gave informed consent before randomisation to usual care given by the Continence/Physiotherapy Service (the control group) or usual care plus use of the IncoStress device (the intervention group).

Randomisation was computer generated and managed by South East Wales Trials Unit (SEWTU) using sealed envelopes in blocks of nine, so that allocation to intervention and control was in the ratio of 2:1 in favour of the intervention group to gain as much information as possible on response to the intervention.

**Fig. 1:**
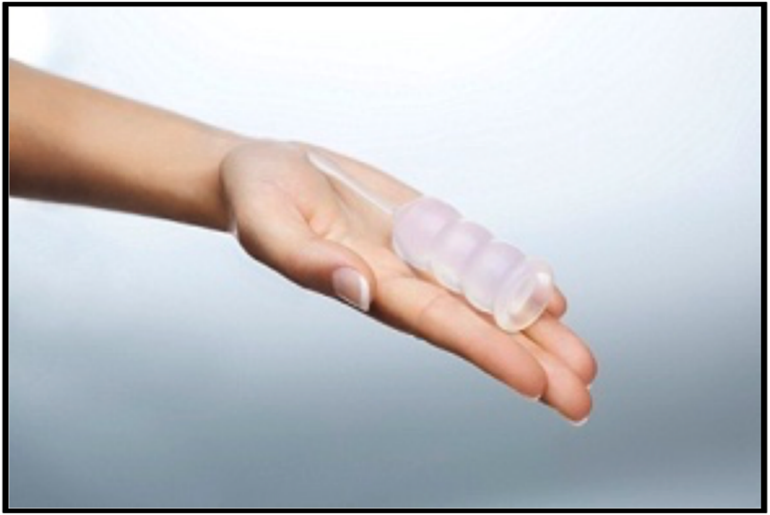
IncoStress device

The intervention group received the IncoStress. This is a silicone tampon-shaped intravaginal device with a tail to facilitate removal. It is reusable, easy to use and clean. It has a retail value of £30 per item and is widely available via internet sites (Fig. 1).

The IncoStress device was chosen for this study because it is a simple and easy-to-use device and there are few published data on clinical and cost effectiveness. Collection of such data would provide useful evidence about similar mechanical devices. Several devices exist but none appear to have been widely adopted.

The treatment period was for 6 months and assessments were carried out at baseline (prior to randomisation), 3 and 6 months post-randomisation. Data were collected via quantitative self-completion questionnaires, using validated scales. The inclusion and exclusion criteria for this study are demonstrated in Box 1. The exclusion criteria were women who were unsuitable for primary conservative management or for use of a mechanical device or if they had any of the conditions listed in Box 1.

Box 1 Inclusion and exclusion criteria

**Table:** 

Inclusion criteria	
Women over the age of 18 years	
Women with symptoms of urge, stress or mixed incontinence attending the Continence Service or the Women’s Health Physiotherapy Service	
Exclusion criteria	
Current medical history of microscopic/macroscopic haematuria	
Recurrent or persistent urinary tract infection (UTI) (two or more UTI’s treated in the preceding 6 months)	
Identified pelvic mass	
Moderate or severe prolapse (stages 3 and 4)	
Palpable bladder	
Bladder or urethral pain	
Possible neurological problem	
Possible urogenital fistula	
Previous radiotherapy or surgery for pelvic cancer	
Symptoms of voiding difficulty	
Pregnancy or intention to get pregnant during the study period	
Inability to use the device due to either physical or mental impairment, including severe atrophic vaginitis or complete lack of pelvic tone (grade 0 on the modified Oxford Scale)	
Vaginal or urinary infection (these women will be eligible once the infection has been treated)	
Known allergy or sensitivity to silicone	

Data collected included demographic information, body mass index, urinary symptoms, usage of incontinence pads, practice of pelvic floor exercises and/or bladder training before recruitment and at each follow-up.

Process outcomes of recruitment, retention and compliance with treatments were recorded. User outcomes were also noted via a questionnaire and included: satisfaction with using IncoStress, the time of usage and reasons to stop using the device as well as acceptability of using it during activities such as exercise, gardening, social events, shopping and housework.

The disease-specific outcomes were assessed: the Incontinence Quality of Life scale (IQOL) and the Female Lower Urinary Tract Symptoms (ICIQ-FLUTS) questionnaire at baseline and follow-up [10, 11].

Both questionnaires have been validated. The IQOL goes from a 0 to 100 score; the higher the score is the better quality of life. In the case of the ICIQ-FLUTS, the higher the score the worse the symptoms are.

Data were analysed using SPSSv22 and presented as median (range) or number (%). No formal statistical between-group comparisons were carried out because the study was designed as a feasibility study and so sample size was determined to allow assessment of recruitment and retention, as well as acceptability of the device and research methods. A sample size calculation was not, therefore, made on the basis of any of the disease specific outcome measures.

A sample of ten participants was invited to take part in a qualitative interview during the final 3 months of the study. This was carried out to better understand views regarding frequency and ease of use of the device as well as overall satisfaction and recommendations for changes to the research processes, which could be incorporated into a future large multi-centre trial.

## Results

Eighty participants were recruited between October 2011 and January 2015 (51 intervention, 29 control). Median age was 45 years (27–70) and median BMI was 26.4 kg/m² (16.5–43.8).

**Fig. 2:**
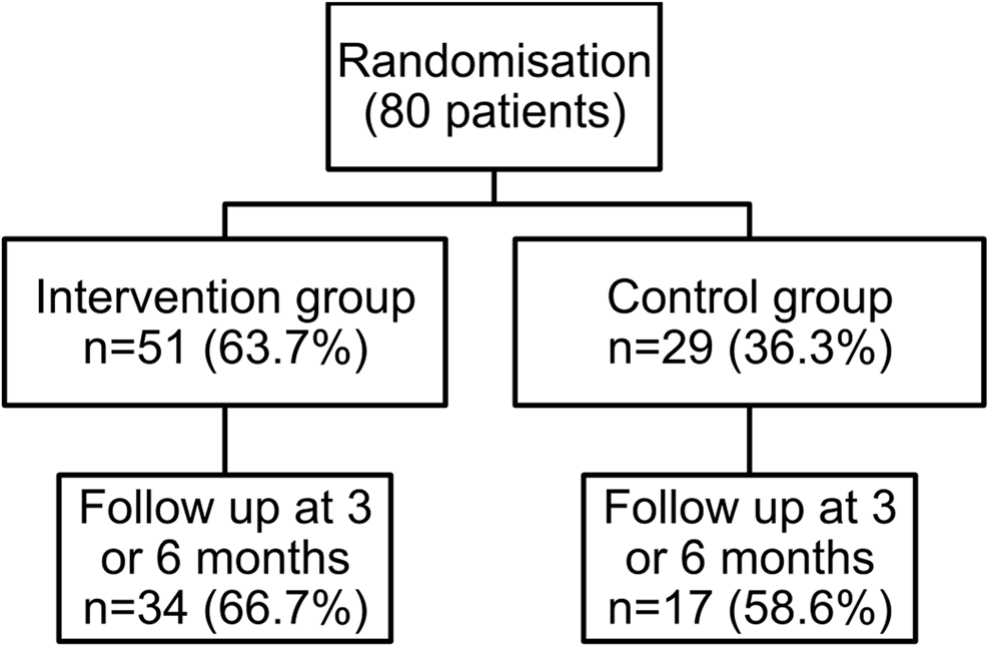
Study flowchart

Follow-up responses were obtained from 34 intervention group patients (66.7%) and 17 (58.6%) controls (Fig. 2). Due to the logistic difficulty of respondents requiring several reminders to return questionnaires, the distinction in the timing of the return to the 3 and 6 month questionnaire was not clear. Therefore, the follow-up data were pooled into a combined outcome assessment. However it can be mentioned that the response rate at 3 months from the intervention group was higher than at 6 months, which could suggest better retention of this group in the short term. In contrast, the response rate from the control group at 3 and 6 months was similar.

Of the 34 participants from the intervention group who completed the incontinence questionnaire (ICI), 4 complained of SUI (11.8%), 1 had UUI (2.9%) and 22 reported MUI (64.7%). Seven did not answer this question (20.6%).

Twenty-two of the 34 women from the intervention group used the device, for a median 3 days a week (0–7), 7 h a day (0–12). Of these, 22 patients (64.7%) reported no vaginal discomfort, 18 (53%) found it easy to use and 21 (61.8%) were satisfied with the device.

Baseline and follow-up data are presented in Table 1.

**Table 1:** Demographic data, pelvic floor exercise, bladder training, IQOL and ICIQ-FLUTS score at baseline/follow-up

Factor	Intervention (51)		Control (29)	
	Baseline	Follow-up	Baseline	Follow-up
Age (years)	44 (27–68)		48 (28–70)	
BMI kg/m²	26.2(20–44)		29.0 (17–43)	
PFE	41 (80%)	29 (85%)	22 (76%)	15 (88%)
BT	22 (43%)	18 (53%)	16 (55%)	6 (35%)
IQOL (0–100)	42.4 (0–94)	68.2 (5–98)	45.5 (0–88)	53.0 (0–94)
ICIQ-FLUTS (0–48)	14.5 (6–35)	12.5 (4–26)	15.0 (5–35)	14.0 (6–38)

*PFE* pelvic floor exercises, *BT* bladder training, *IQOL score* urinary incontinence quality of life scale, *ICIQ-FLUTS score* female lower urinary tract symptoms and impact on quality of life

At baseline, the percentage of participants performing PFE was similar between the two groups. The percentage of people practising bladder training was slightly less (Table 1).

At follow-up, there was no difference in the percentage of participants performing PFE or bladder training (Table 1).

Regarding disease-specific outcomes, the median IQOL score in the intervention group improved from a baseline of 42.4 (0–94) to 68.2 (5–98) at follow-up and in the control group from baseline 45.5 (0–88) to 53.0 (0–94). The median ICIQ-FLUTS score in the intervention group improved from 14.5 (6–35) to 12.5 (4–26) and in the control group from 15.0 (5–35) to 14.0 (6–38) (Table 1).

No serious adverse events were noted throughout the study. Four patients from the intervention group had a urinary tract infection while using the device and one had a vaginal infection. Those patients were treated by the GP.

Ten interviews were carried out with women between the ages of 33 and 78 years. Most participants found the device easy to use and clean. Two reported difficulties with the device falling out, so they used it more during the night. Most participants reported that they would be prepared to pay around £30 for the device as it had improved their quality of life. Eight would recommend the device to others, suggesting it could prevent further invasive treatment. Some of the quotes by patients were: *‘IncoStress has helped over the past 6 months. I am glad I have taken part in this experiment, as it has shown me that there are things that can help, before surgery needs to be considered’. ‘Took a while to get used to it. I have noticed a big difference and feel a lot more confident, but I still have to wear a sanitary towel. I have had accidents when I have a cold/cough’ and ‘Unable to use. Will not stay in’.*

## Discussion

This study suggests improvements in quality of life and urinary symptoms after usage of the IncoStress device compared with the control group who received standard care and advice on pelvic floor exercises and/or bladder retraining. This may imply that the IncoStress device is effective; however a larger sample and statistical analysis are required to confirm it. Similar findings were also demonstrated in another RCT, published by Cornu et al. in 2012 [12]. They reported that the 75NC007 intravaginal device was effective in decreasing the number of episodes of SUI in 29 patients and improving the global score of quality of life. These results indicate that intravaginal devices have a place in the conservative management of urinary incontinence, especially in those patients who do not wish to have surgical intervention or are not fit for it or whilst awaiting surgery, although a large, well-designed study would confirm this. Devices may also be useful for those who have not completed their family or wish to stop leakage whilst doing exercises [13].

In our study, most of the patients used the device during the day, finding it acceptable, and overall easy to use and of those who used it most were satisfied. Equally, Cornu et al. reported that the 75NC007 intravaginal device was found to be easy to use (26 out of 46) and acceptable by most of the patients (38 out of 46), although women were not asked about satisfaction with the device [12]. In our study, some patients used the device mainly at night because it did not stay in place whilst doing any activities and some found it uncomfortable. It is likely that mechanical devices are not suitable for everyone so further work on patient selection, support and education from practitioners is important in the design phase of a large study. This is to avoid loss to follow-up and low satisfaction while the device is being tested.

Symptom response was significant, but there was loss to follow-up. Our dropout rate was similar to Thyssen et al.'s study (34%) [13]. In our study, not all participants provided 3- and 6-month questionnaire data, so the data that have been obtained and analysed are the data they provided. For that reason we pooled the outcome as a single response. This could be improved, however, using a retention strategy in a better-resourced larger study.

Our results suggest that recruitment to a study of intravaginal devices is feasible and randomisation processes in this case were robust. This indicates that a larger multicentre RCT would be possible in the near future. We recognised that recruitment was not rapid. This was due to local staffing issues, especially lack of research nurses to recruit and follow-up patients and in some way the reliance on clinical staff to recruit. We believe that the relatively high attrition rate was a consequence of the staffing issues rather than fundamental difficulties with the study design. If a study were funded fully, this would not be such a problem. Unfortunately, we were unable to calculate the recruitment rate as the study took place over such an extended period of time at several sites and the number of participants approached was not recorded. However, the recruitment rate varies from study to study, between 21 and 75% [14].

Research on mechanical devices for UI is limited, although studies that are available do show some positive results [12, 13, 15, 16]. Although devices present a potentially cheap intervention and give immediate relief of symptoms, they are not widely used because the evidence is not available on clinical or cost effectiveness or for whom and under what circumstances they may be most beneficial.

There are few qualitative data concerning the acceptability or support needs when using devices. In addition, previous studies have tended to use clinical outcome measures rather than quality of life as the primary outcome. A Cochrane reviewed published in 2014 concluded that there is little evidence from controlled trials on whether mechanical devices are better than no treatment or if one device was better than another. A total of eight trials were reviewed. Three trials compared intravaginal mechanical devices with no treatment where they concluded that the use of a mechanical device might be better than no treatment although the evidence for this was inconclusive. Other trials compared one mechanical device with another; three compared intra-urethral devices and two intravaginal devices, but quantitative synthesis of data from these trials was not possible because different mechanical devices were compared in each trial using different outcomes [17]. It is also not known what role such devices play within the framework of guidelines for primary treatments as the manufacturer of the device recommends that it be used in conjunction with pelvic floor therapy.

Published data and this pilot suggest devices are a useful adjunct to conservative treatment packages for women with mixed and stress urinary incontinence. This pilot demonstrates the potential value of IncoStress and confirms the feasibility of a larger randomised controlled trial of the effectiveness of vaginal devices for urinary incontinence. A further longer-term randomised controlled trial comparing different mechanical devices with a large sample should be carried out to fully evaluate this device and define the role in care.

Recruitment was feasible and randomisation processes were robust. Symptom response was significant but follow-up requires some attention to keep losses to a minimum.

## Abbreviations

UI: Urinary incontinence
IQOL questionnaire: Urinary incontinence quality of life scale
ICIQ-FLUTS questionnaire: Female lower urinary tract symptoms and impact on quality of life
RCT: Randomised controlled trial
SEWTU: South East Wales Trials Unit
UTI: Urinary tract infection

## References

[1] Minassian VA, Stewart WF, Wood GC (2008). Urinary incontinence in women: variation in prevalence estimates and risk factors. Obstet Gynecol.

[2] Turner DA, Shaw C, McGrother CW, Dallosso HM, Cooper NJ, Turner NJ (2004). The cost of clinically significant urinary storage symptoms for community dwelling adults in the UK. BJU Int.

[3] Smith A, Bevan D, Douglas HR, James D. Management of urinary incontinence in women: summary of updated NICE guidance. BMJ Br Med J. 2013;347. 10.1136/bmj.f5170 24021756

[4] Richter EH, Burgio LK, Brubaker EL, Nygaard SI, Ye LW, Weidner SA (2010). Continence pessary compared with behavioral therapy or combined therapy for stress incontinence: a randomized controlled trial. Obstet Gynecol.

[5] Kenton K, Barber M, Wang L, Hsu Y, Rahn D, Whitcomb E (2012). Pelvic floor symptoms improve similarly after pessary and behavioral treatment for stress incontinence. Female Pelvic Med Reconstr Surg.

[6] Clemons JL, Aguilar VC, Tillinghast TA, Jackson ND, Myers DL (2004). Patient satisfaction and changes in prolapse and urinary symptoms in women who were fitted successfully with a pessary for pelvic organ prolapse. Obstet Gynecol.

[7] Ding J, Chen C, Song X, Zhang L, Deng M, Zhu L (2016). Changes in prolapse and urinary symptoms after successful fitting of a ring pessary with support in women with advanced pelvic organ prolapse: a prospective study. Urology.

[8] Al-Shaikh G, Syed S, Osman S, Bogis A, Al-Badr A. Pessary use in stress urinary incontinence: a review of advantages, complications, patient satisfaction, and quality of life. Int J Women's Health. 2018:195–201. 10.2147/IJWH.S152616 PMC5909791 29713205

[9] Kielb SJ (2005). Stress incontinence: alternatives to surgery. Int J Fertil Womens Med.

[10] Avery K, Donovan J, Peters TJ, Shaw C, Gotoh M, Abrams P (2004). ICIQ: a brief and robust measure for evaluating the symptoms and impact of urinary incontinence. Neurourol Urodyn.

[11] Wagner TH, Patrick DL, Bavendam TG, Martin ML, Buesching DE (1996). Quality of life of persons with urinary incontinence: development of a new measure. Urology.

[12] Cornu J, Mouly S, Amarenco G, Jacquetin B, Ciofu C, Haab F (2012). 75NC007 device for noninvasive stress urinary incontinence management in women: a randomized controlled trial. Int Urogynecol J.

[13] Thyssen H, Bidmead J, Lose G, Møller Bek K, Dwyer P, Cardozo L (2001). A new intravaginal device for stress incontinence in women. BJU Int.

[14] Donnelly M, Powell-Morgan S, Olsen A, Nygaard I (2004). Vaginal pessaries for the management of stress and mixed urinary incontinence. Int Urogynecol J.

[15] Shaikh S, Ong EK, Glavind K, Cook J, N'Dow JM. Mechanical devices for urinary incontinence in women. Cochrane Database Syst Rev. 2011;12(7):CD001756. 10.1002/14651858.CD001756.pub5 21735385

[16] Bonnar J (1977). Silicone vaginal appliance for control of stress incontinence. Lancet.

[17] Lipp A, Shaw C, Glavind K. Mechanical devices for urinary incontinence in women. Cochrane Database Syst Rev. 2014(Issue 12. Art. No.: CD001756. 10.1002/14651858.CD001756.pub6. 10.1002/14651858.CD001756.pub6 PMC7061494 25517397

